# Eligibility for Isoniazid Preventive Therapy in South African Gold Mines

**DOI:** 10.1371/journal.pone.0081376

**Published:** 2013-11-14

**Authors:** James J. Lewis, Katherine L. Fielding, Alison D. Grant, Violet N. Chihota, Flora Popane, Mariette Luttig, Dorothy Muller, Leonie Coetzee, Gavin J. Churchyard

**Affiliations:** 1 London School of Hygiene and Tropical Medicine, London, United Kingdom; 2 The Aurum Institute for Health Research, Johannesburg, South Africa; National Institute of Allergy and Infectious Diseases, United States of America

## Abstract

**Setting:**

The “Thibela TB” cluster randomised trial of community-wide isoniazid preventive therapy (IPT) to reduce tuberculosis incidence in the South African gold mines.

**Objectives:**

To determine the proportion of participants eligible for IPT and the reasons and risk factors for ineligibility, to inform the scale-up of IPT.

**Design:**

Cross-sectional survey of participants in intervention clusters (mine shafts) consenting to tuberculosis screening and assessment for eligibility to start IPT.

**Results:**

Among 27,126 consenting participants, 94.7% were male, the median age was 41 years, 12.2% reported previous tuberculosis, 0.6% reported ever taking IPT and 2.5% reported currently taking antiretroviral therapy. There were 24,430 (90.1%) assessed as eligible to start IPT, of whom 23,659 started IPT. The most common reasons for ineligibility were having suspected tuberculosis that was subsequently confirmed by a positive smear and/or culture (n=705), excessive alcohol consumption (n=427) and being on tuberculosis treatment at time of initial screen (n=241). Ineligibility was associated with factors including older age, female gender, prior history of tuberculosis and being in “HIV care”. However, at least 78% were eligible for IPT in all of these sub-groups.

**Conclusions:**

The vast majority of participants in this community-wide intervention were eligible for IPT.

## Introduction

Settings with a high prevalence of the human immunodeficiency virus (HIV) have experienced dramatic increases in tuberculosis incidence [1]. Alternative approaches are necessary to reduce the risk of HIV associated tuberculosis in such settings. Approaches promoted by the World Health Organization (WHO) include antiretroviral therapy and its 3Is strategy of isoniazid preventive therapy (IPT), intensified case finding (ICF) and infection control [1]. IPT was provided to almost 450,000 newly enrolled into HIV care in 2011, up from 201,000 in 2010, yet still far lower than the total number newly enrolled in HIV care and potentially eligible for IPT (estimated at 1.5 million in 2010) [1]. In 2011, the WHO released simplified guidelines for IPT and ICF, recommending that all people living with HIV should be regularly screened for tuberculosis using a clinical algorithm of any of cough, night sweats, weight loss and/or fever. Those without any of these symptoms and without further contraindications to IPT should be enrolled on IPT [2]. The Global Plan to Stop TB (2011-2015) has set a target of universal IPT coverage for all eligible persons living with HIV by 2015, assuming that half of all people living with HIV will be eligible [3]. However, the actual percentage that would be eligible for IPT under these new guidelines it is not known and this has clear implications for the required scale-up and subsequent impact.

The South African gold mines have experienced a dramatic increase in tuberculosis case notifications, to greater than 4% per year [4], caused in part by high prevalences of HIV (29%) and silicosis [5,6]. This has happened despite implementation of the World Health Organization’s DOTS (Directly Observed Therapy, Short course chemotherapy) strategy and routine radiological screening for tuberculosis. “Thibela TB” is a cluster randomised study, in the South African gold mines, evaluating the impact of community-wide tuberculosis screening followed by IPT, in addition to standard of care, on tuberculosis incidence [7]. Understanding the eligibility profile of those who consented to be screened for suitability to start IPT as part of the Thibela TB intervention will thus inform both future roll-outs of IPT and contextualize the results of the study. The aim of this analysis was to determine the proportion, reasons and factors associated with ineligibility among those who consented to Thibela TB.

## Methods

### Ethics Statement

"Thibela TB" was approved by the Research Ethics Committees of the University of KwaZulu Natal and the London School of Hygiene and Tropical Medicine. All consenting participants gave written consent or, for illiterate participants, witnessed oral consent. For illiterate participants, there was an impartial witness present during the consenting process, who then signed the relevant witness section of the consent form. Both ethics committees approved the consent form, including the section on the use of witnessed oral consent for illiterate participants, at the beginning of the study.

### Setting: Thibela TB study

The Thibela TB study has been described in detail elsewhere [7]; briefly, all consenting workers in the eight intervention clusters were screened for tuberculosis and, if eligible, received nine months of IPT (300mg daily, self-administered, plus pyridoxine 25mg daily). All employees and contractors were invited to take part in the study, regardless of HIV or silicosis status and of previous history of tuberculosis or IPT. The three mining companies taking part in Thibela TB provide free tuberculosis and HIV care to all employees. 

### Intervention and screening for eligibility

The intervention was designed to be delivered by trained nurses with eligibility determined on clinical grounds as far as possible and laboratory tests only conducted for suspected tuberculosis and suspected hepatitis. Permanent ineligibility criteria (as summarised in Table 1) were: currently on tuberculosis treatment or IPT; known or suspected hypersensitivity to isoniazid; chronic liver disease; excessive alcohol use (defined as exceeding 28 units per week for men or 21 units per week for women; note one unit is equivalent to 10ml of pure alcohol); history of convulsions or psychosis; peripheral neuropathy grade 2 (moderate) or greater, as defined by the AIDS clinical trials group [8]; measured weight less than 40 kg; a woman who was pregnant (by self-report or urine test), or up to 3 months post-partum, or of child bearing potential and declined to use contraception, or planned to become pregnant over the next 12 months; receipt of an investigational drug or product within the previous 30 days; or receipt of contraindicated drugs (defined as any of: carbamazepine and phenytoin; selective serotonin reuptake inhibitor antidepressants; disulfiram; warfarin; theophylline; oral ketoconazole or itraconazole). 

**Table 1:** Eligibility criteria for isoniazid preventive therapy, contrasting those for the Thibela TB study to those of the guidelines from the World Health Organization in 2011 [2] and the South African Department of Health in 2010 [12].

**Eligibility**	**Thibela TB**	**World Health Organization 2011**	**South African Department of Health 2010**
HIV status	No restriction	HIV infected only	HIV infected only
TB screen	Investigate for TB if any of: cough > two weeks, night sweats, weight loss, other compatible symptom, compatible chest X-ray abnormality	Evaluate for TB if any of: current cough, fever, weight loss, night sweats, “chest radiography can be done if available”	Investigate for TB if any of: current cough, fever, weight loss, night sweats
ART	No restriction	No restriction	No restriction
Prior history of TB	No restriction	No restriction	No restriction
Pregnancy	Excluded if any of: pregnant, up to 3 months post-partum, of child bearing potential and declined to use contraception, or planned to become pregnant over the next 12 months	No restriction	No restriction
TST ^1^	Not done	Unnecessary	Unnecessary ^2^
Hepatitis	Chronic liver disease or hepatitis suspect confirmed by liver function tests	Active hepatitis (acute or chronic)	Active liver disease
Alcohol exclusion	>28 units per week for men, >21 units per week for women	Regular and heavy alcohol consumption	“Actively abusing alcohol”
Other contra-indications	History of convulsions or psychosis; peripheral neuropathy grade 2 (moderate) or greater; weight <40 kg; receipt of an investigational drug or product within the previous 30 days; or contraindicated drugs	Symptoms of peripheral neuropathy	None specified

1 TST = tuberculin skin test

2 The 2013 South African antiretroviral treatment guidelines state that IPT should be provided regardless of TST result, but contain revised guidance that a TST is now required before offering at least 36 months of IPT [18].

Participants with no reasons for permanent ineligibility were classified as temporarily ineligible if: they were tuberculosis or hepatitis suspects (defined below); had a rash consistent with hypersensitivity, to ensure that those starting IPT were not unnecessarily stopped due to a pre-existing rash; or were women of child bearing potential that were required to start effective contraception prior to starting IPT. Participants who were temporarily ineligible were reassessed at a review visit and then reclassified as permanently ineligible or eligible to start IPT, depending on the results of tuberculosis or hepatitis investigations or whether they had started contraceptive use.

All screening was performed by trained study nurses and chest radiographs were read by experienced radiographers.

### Definitions

The Thibela TB intervention was designed in 2004, pre-dating the 2011 WHO guidelines on screening for tuberculosis prior to IPT [2], and so the development of the screening algorithm was based on our previous tuberculosis screening work in a gold mining population [5]. Hence, participants were defined as tuberculosis suspects if they had one or more of: cough for two weeks or longer, drenching night sweats, unintentional weight loss, another symptom that the study nurse thought could be due to tuberculosis or a chest radiographic abnormality suggestive of active tuberculosis. If the chest radiographic abnormality was suggestive of old tuberculosis, the study chest radiograph was compared with a previous one taken within the past two years (as part of the routine annual medical examination for fitness for work) and the participant was asked to return in one week. If the chest radiographic lesions were new or changing, or a previous chest radiograph could not be found, the participant was investigated as a tuberculosis suspect. 

Tuberculosis suspects were investigated by collecting one spot sputum for flurochrome microscopy and Mycobacterial Growth Indicator Tube (MGIT) culture. Positive cultures were confirmed for the presence of mycobacteria by examination of Ziehl-Neelsen stained slides for acid fast bacilli and for the presence of *Mycobacterium tuberculosis* using the anti MPB64 monoclonal antibody lateral flow assay (TAUNS, Numazu, Japan). Those who were smear or culture positive and those with unresolved symptoms at the follow up visit were referred to the routine mine health services for further investigations and were permanently ineligible for IPT in the study.

A hepatitis suspect was defined as a participant with any of the following: nausea or vomiting in the past week, at Division of AIDS grade 2 (moderate) or above [8]; right upper quadrant pain in the past week; urine darker than normal in past 48 hours; or jaundice (self-reported or on examination). Hepatitis suspects were then referred to the health service for liver function tests and reviewed four weeks later with the results. Participants were ineligible for IPT if at this review they reported any of: nausea or vomiting in the past 24 hours; right upper quadrant pain in the past week; urine darker than normal over past 48 hours; eyes had gone yellow recently (and the study nurse agreed this was the case); a medical doctor had told the participant they had a liver problem in the last four weeks; or elevated liver function tests (defined as any of: total bilirubin >5 times upper limit of normal [ULN]; alkaline phosphatase >10 x ULN; AST >2.5 times ULN; ALT >2.5 times ULN).

HIV testing was not part of the study. A proxy variable of “HIV care” combined those reporting being currently on antiretroviral therapy with those reporting previous IPT use (because targeted IPT was delivered by mine health services to HIV clinic attendees, with variable coverage, and rarely used otherwise). This variable will inevitably have underestimated those who genuinely had HIV; conversely, pilot work showed that the great majority of people who reported taking ART or IPT gave correct information. This variable thus had low sensitivity but high specificity for documented HIV infection. Current IPT use was an exclusion criterion for the Thibela TB study and so was not part of this variable.

### Data collection and analysis

Data were directly entered into a proprietary database (InForm, Oracle, California), with appropriate range limits, validation checks and skip patterns. Data management and analysis used STATA v.11 (Stata Corporation, College Station, Texas). Enrolment to the intervention took place in two phases: during the first phase all employees and contractors working in the cluster were eligible for recruitment and were encouraged to attend the study centre during this time period; this was followed by a nine month phase to allow all individuals who had started IPT to complete their nine month course, during which time only new recruits to the workforce were eligible for enrolment (second phase). All those who consented to take part in Thibela TB during this first phase of recruitment were included in this analysis. All confidence intervals quoted were exact.

## Results

Between July 2006 and May 2009, 27,126 individuals consented to take part in the Thibela TB study in the eight intervention clusters. Reflecting the general gold mining workforce, they were 94.7% male, median age 41 years (inter-quartile range: 32-47 years), median years of employment 16 years (inter-quartile range: 5-25 years), 98.1% were black, 59.5% were South African and 55.4% lived in hostels (Table 2). 12.2% reported previous tuberculosis, 0.6% reported ever taking IPT and 2.5% reported currently being on antiretroviral therapy. 

**Table 2:** Proportion ineligible for isoniazid preventive therapy in sub-groups (n=26,912).

	**Consented to screen**		**Ineligible**	
	**N**	**Column %**	**N**	**Row % (95% CI)**
**Age group (years)**				
<35	8,370	31.1%	594	7.1% (6.6%, 7.7%)
35-44	9,091	33.8%	826	9.1% (8.5%, 9.7%)
45-54	8,062	30.0%	998	12.4% (11.7%, 13.1%)
55+	1,389	5.2%	207	14.9% (13.1%, 16.9%)
**Sex**				
Male	25,493	94.7%	2,409	9.4% (9.1%, 9.8%)
Female	1,419	5.3%	216	15.2% (13.4%, 17.2%)
**Country of origin**				
SA	16,063	59.7%	1,605	10.0% (9.5%, 10.5%)
Lesotho	6,716	25.0%	745	11.1% (10.4%, 11.9%)
Mozambique	2,953	11.0%	162	5.5% (4.7%, 6.4%)
Other	1,180	4.4%	113	9.6% (8.0%, 11.4%)
**Years in workforce**				
0-9	9,277	34.5%	710	7.7% (7.1%, 8.2%)
10-19	6,611	24.6%	551	8.3% (7.7%, 9.0%)
20-29	7,340	27.3%	812	11.1% (10.4%, 11.8%)
30+	3,684	13.7%	552	15.0% (13.8%, 16.2%)
**Ethnic group**				
Black	26,393	98.1%	2,565	9.7% (9.4%, 10.1%)
Other	519	1.9%	60	11.6% (8.9%, 14.6%)
**Place of residence**				
Hostel/family unit	14,841	55.1%	1,438	9.7% (9.2%, 10.2%)
Informal housing	1,930	7.2%	203	10.5% (9.2%, 12.0%)
Single quarters/other	652	2.4%	55	8.4% (6.4%, 10.8%)
House	9,489	35.3%	929	9.8% (9.2%, 10.4%)
**Hx of tuberculosis**				
No	23,625	87.8%	1,914	8.1% (7.8%, 8.5%)
Yes	3,287	12.2%	711	21.6% (20.2%, 23.1%)
HIV care *				
No	26,122	97.1%	2,458	9.4% (9.1%, 9.8%)
Yes	790	2.9%	167	21.1% (18.3%, 24.2%)
Weight (kg) **				
41-60	4,964	18.4%	713	14.4% (13.4%, 15.4%)
61-80	16,201	60.2%	1,489	9.2% (8.8%, 9.6%)
>80	5,747	21.4%	423	7.4% (6.7%, 8.1%)
Alcohol use (units/week)***				
0	15,427	57.3%	1,295	8.4% (8.0%, 8.8%)
1-28	11,061	41.1%	906	8.2% (7.7%, 8.7%)
29+	424	1.6%	424	100.0%

CI = confidence interval

* “HIV care” is a proxy from having ever taken IPT or currently being on ART

** Weight less than or equal to 40kg was an exclusion criteria, but none of the participants in this table were excluded on this basis.

*** for women grouping is 0, 1-21, 22+. Excessive alcohol use, defined as 29+ units/week for men and 22+ units/week for women, was an exclusion criterion; hence, no confidence interval was given for the proportion.

Note: 214 participants had missing data on at least one of these variables and so were excluded from this analysis; they were more likely to be ineligible than those with no missing data (33.2% versus 9.8% respectively, p<0.001).

At the enrolment visit, 1,249 of the 27,126 (4.6%) who consented were classified as permanently ineligible, 3,111 (11.5%) as temporarily ineligible and 22,766 (83.9%) were immediately eligible to start IPT. The most common reasons for being classified as permanently ineligible were: excessive alcohol consumption (427, 1.6%); being on tuberculosis treatment (241, 0.9%); contraindicated medications (226, 0.8%); history of convulsions (136, 0.5%); pregnancy, planning on becoming pregnant or unwillingness to use contraceptives while taking IPT (107, 0.4%); all other reasons were given by fewer than fifty participants, including those already on IPT (47, 0.2%). Participants with missing data (48, 0.2%) were considered as ineligible. 

1,879 of 27,126 (6.9%) participants were classified as tuberculosis suspects, based on symptom screen or radiographic abnormalities, regardless of other reasons for ineligibility. Of these suspects, 665 were culture positive (35.4% of all tuberculosis suspects), of whom 343 (51.6%) were MPB64 positive for *Mycobacterium tuberculosis* and two did not have the test done. An additional 40 were culture negative (or contaminated) and smear positive. Among the 383 who were culture positive for *M. tuberculosis* or smear positive, only 150 (39.2%) were symptomatic, with the remaining 233 investigated because of compatible abnormalities on their chest radiograph. A further 1,281 participants were temporarily ineligible while their current chest radiograph was compared to a previous one, but were then classified as not having evidence of tuberculosis i.e. were not classified as tuberculosis suspects. 

There were 126 participants initially classified as temporarily ineligible for reasons other than tuberculosis, regardless of other reasons for ineligibility: 58 had rash consistent with hypersensitivity, 40 were temporarily ineligible while reliable contraception was arranged, 12 had symptoms suggesting hepatitis and 20 for other reasons. 55 of the 126 (43.7%) returned for their review visit, at which 10 were reclassified as permanently ineligible (7 due to unresolved rash consistent with hypersensitivity, 2 had not started contraceptive use and 1 due to both of these). 

In total, 24,430 of the 27,126 (90.1%) who consented were finally classified as eligible to start IPT, of whom 23,659 started IPT (87.2% of all those who consented). There were 971 tuberculosis cases (positive smear and/or culture, on tuberculosis treatment at screen or starting tuberculosis treatment while temporarily ineligible), while a further 734 were ineligible due to suspected tuberculosis. Ineligibility was associated with the following factors: older age, female gender, non-Mozambican, with longer duration in the workforce, prior history of tuberculosis, in “HIV care” and of lower weight (Table 2). However, even among those with prior history of tuberculosis, the sub-group with the highest percentage ineligible, 78.4% (95% confidence interval: 76.9%, 79.8) were eligible for IPT (Table 2). 

## Discussion

This study found that 90.1% of participants screened were eligible to start IPT. The commonest reason for ineligibility was a positive smear and/or culture (705 of 27,126 consenting [2.6%]), underscoring the importance of tuberculosis screening prior to starting IPT.

The eligibility criteria in “Thibela TB” were deliberately wide as the safety profile of isoniazid is well characterised and the risk of tuberculosis in this mining population is very high in all subgroups [5,9-11]. There were no restrictions on IPT use with antiretroviral therapy, previous tuberculosis treatment, previous IPT use, silicosis, age or moderate alcohol use, consistent with the 2011 WHO and 2010 South African Department of Health guidelines for IPT (Table 1) [2,12]. Despite these wide eligibility criteria, and the use of self-report for initial assessments rather than laboratory tests or medical record review, the safety profile of isoniazid in this nurse-delivered study was good [13], validating the choice of criteria to maximise the uptake of IPT.

In this analysis, differences in ineligibility between sub-groups were due to these groups acting as proxies for exclusion criteria. The factors associated with ineligibility were similar to those associated with tuberculosis [9], such as for increasing age and years in the workforce, country of origin, previous tuberculosis and being in HIV care. This was unsurprising as active tuberculosis made up a substantial proportion of the reasons for ineligibility. In contrast, women were more likely to be ineligible, despite being at lower risk for undiagnosed tuberculosis, due to the exclusion criteria of pregnancy, planning on becoming pregnant or unwillingness to use contraceptives. 

Eligibility in this study was higher than observed in studies screening for IPT among HIV-infected adults [14-17]. A randomised trial comparing six months versus 36 months of IPT among adults accessing antiretroviral therapy in Botswana used a two-stage screening process [15]. The first stage applied the exclusion criteria from the Botswana National IPT programme to 4,018 consenting participants; this excluded 27% of those screened, predominantly due to illness, recent history of tuberculosis and prior IPT (66%, 9% and 8% respectively). The second stage applied study specific exclusion criteria to the remaining 2,934 participants; this excluded a further 776 (26%), most commonly due to chest radiographs compatible with tuberculosis (296 of 776 [36%]). Another randomised trial comparing six months IPT to 36 months isoniazid and ethambutol screened 1,095 HIV-infected persons in India, of whom 268 (24.5%) were excluded for study-specific and 144 (13.2%) for non-study specific criteria (29 culture positive, 115 other unspecified criteria) [16]. A randomised trial of four preventive regimens screened 1,528 tuberculin-positive, HIV-infected adults in South Africa, of whom 324 were ineligible (21.2%), including 141 (9.2% of 1528) who would be ineligible under current guidelines (90 active tuberculosis, 39 chest radiograph compatible with tuberculosis, 4 on tuberculosis treatment and 8 other) [17]. A retrospective evaluation of intensified tuberculosis case finding and IPT among newly diagnosed HIV infected adults in a Voluntary Counselling and Testing clinic in Uganda found 5% had active tuberculosis and a further 37% were excluded from IPT, predominantly because of distance from the clinic, stage 4 HIV disease and previous history of tuberculosis (45%, 30% and 27% respectively) [14]. Under the wider eligibility criteria of “Thibela TB”, many of those excluded in these other studies would have been eligible for IPT, either immediately or following further tuberculosis investigations. In addition, populations with relatively high proportions of people with later stage HIV disease, where it is more difficult to exclude active tuberculosis, are likely to have a higher proportion ineligible. As HIV testing is scaled up and individuals are enrolled into care at earlier stages of HIV disease, the proportion eligible for IPT may increase as the prevalence of symptoms and undiagnosed tuberculosis will be lower. 

The generalisability of these results to other settings were IPT is being rolled out at scale may be thought to be limited, as the study investigated community-wide rather than the more common approach of targeted IPT, and was conducted in the mining industry with a predominance of men and possibly a “healthy worker effect”. However, although this strategy of community-wide IPT, which was conducted irrespective of HIV status, is more likely to find healthy, asymptomatic people than targeting HIV infected persons, the scale up of HIV testing is likely to result in increasing proportions of healthy, asymptomatic people entering HIV care. A “healthy worker effect” may result in a population with less severe HIV disease among those who are HIV infected than among those HIV infected in the general population, but this is likely to be true of other settings where community-wide IPT could be implemented, for example, similar workforces and the military. Although women were not well represented in this study, the results presented here show that the vast majority of women were eligible for IPT, which is generalisable. In addition, the main reasons for increased ineligibility among women in this study were the study-specific exclusion criteria of pregnancy or risk of pregnancy. However the 2011 WHO guidelines have made it make clear that these should not be reasons for excluding IPT in a routine setting (Table 1 [2]) and so, eligibility may be higher among women than reported here. Most studies conducted in health care settings have a predominance of women, so one advantage of this study is the large amount of data provided for men.

## Conclusions

This community-wide, nurse-delivered intervention found that the vast majority of individuals assessed were eligible for IPT. As HIV testing is scaled up, particularly community-based testing, and more people with early HIV disease are identified, a high proportion will be eligible for IPT, supporting immediate scale up of HIV testing and IPT.

## References

[1] World Health Organization (2012) Global tuberculosis report. Geneva: World Health Organization.

[2] World Health Organization (2011) Guidelines for intensified tuberculosis case-finding and isoniazid preventive therapy for people living with HIV in resource-constrained settings. Geneva: World Health Organization.

[3] World Health Organization (2010) The Global Plan To Stop TB 2011-2015. Geneva: World Health Organization 10.2471/06.038513 PMC2636638 17639210

[4] Corbett EL, Churchyard GJ, Charalambos S, Samb B, Moloi V et al. (2002) Morbidity and mortality in South African gold miners: impact of untreated disease due to human immunodeficiency virus. Clin Infect Dis 34: 1251-1258. doi:10.1086/339540. PubMed: 11941552. 11941552

[5] Lewis JJ, Charalambous S, Day JH, Fielding KL, Grant AD et al. (2009) HIV infection does not affect active case finding of tuberculosis in South African gold miners. Am J Respir Crit Care Med 180: 1271-1278. doi:10.1164/rccm.200806-846OC. PubMed: 19745207. 19745207 PMC2796737

[6] Corbett EL, Churchyard GJ, Clayton TC, Williams BG, Mulder D et al. (2000) HIV infection and silicosis: the impact of two potent risk factors on the incidence of mycobacterial disease in South African miners. AIDS 14: 2759-2768. doi:10.1097/00002030-200012010-00016. PubMed: 11125895. 11125895

[7] Fielding KL, Grant AD, Hayes RJ, Chaisson RE, Corbett EL et al. (2011) Thibela TB: design and methods of a cluster randomised trial of the effect of community-wide isoniazid preventive therapy on tuberculosis amongst gold miners in South Africa. Contemp Clin Trials 32: 382-392. doi:10.1016/j.cct.2010.12.008. PubMed: 21193066. 21193066

[8] http://rsc.tech-res.com/document/safetyandpharmacovigilance/table_for_grading_severity_of_adult_pediatric_adverse_events.pdf.

[9] Churchyard GJ, Fielding KL, Lewis JJ, Chihota VN, Hanifa Y et al. (2010) Symptom and chest radiographic screening for infectious tuberculosis prior to starting isoniazid preventive therapy: yield and proportion missed at screening. AIDS 24 Suppl 5: S19-S27. doi:10.1097/01.aids.0000391018.72542.46. PubMed: 21079424. 21079424

[10] Corbett EL, Charalambous S, Moloi VM, Fielding K, Grant AD et al. (2004) Human immunodeficiency virus and the prevalence of undiagnosed tuberculosis in African gold miners. Am J Respir Crit Care Med 170: 673-679. doi:10.1164/rccm.200405-590OC. PubMed: 15191919. 15191919

[11] Lewis JJ, Chihota VN, Churchyard GJ, Grant AD, Coetzee L et al. (2008) Factors associated with incident TB in the South African gold mines: a cohort study embedded within Thibela TB (PS-82148-20). 39th World Conference on Lung Health of the International Union Against Tuberculosis and Lung Disease Paris, France.

[12] South African Department of Health (2010) Guidelines for tuberculosis preventive therapy among HIV infected individuals in South Africa. Available: http://hivfshealth.org/document/2010/08/17/guidelines-for-tubercolosis-preventative-therapy-among-hiv-individuals-of-south-.

[13] Grant AD, Mngadi KT, van Halsema CL, Luttig MM, Fielding KL et al. (2010) Adverse events with isoniazid preventive therapy: experience from a large trial. AIDS 24 Suppl 5: S29-S36. doi:10.1097/01.aids.0000391019.10661.66. PubMed: 21079425. 21079425

[14] Mugisha B, Bock N, Mermin J, Odeke RM, Miller B et al. (2006) Tuberculosis case finding and preventive therapy in an HIV voluntary counseling and testing center in Uganda. Int J Tuberc Lung Dis 10: 761-767. PubMed: 16848338. 16848338

[15] Mosimaneotsile B, Mathoma A, Chengeta B, Nyirenda S, Agizew TB et al. (2010) Isoniazid tuberculosis preventive therapy in HIV-infected adults accessing antiretroviral therapy: a Botswana Experience, 2004-2006. J Acquir Immune Defic Syndr 54: 71-77. PubMed: 19934764. 19934764 10.1097/QAI.0b013e3181c3cbf0

[16] Swaminathan S, Menon PA, Gopalan N, Perumal V, Santhanakrishnan RK et al. (2012) Efficacy of a six-month versus a 36-month regimen for prevention of tuberculosis in HIV-infected persons in India: a randomized clinical trial. PLOS ONE 7: e47400. doi:10.1371/journal.pone.0047400. PubMed: 23251327. 23251327 PMC3522661

[17] Martinson NA, Barnes GL, Moulton LH, Msandiwa R, Hausler H et al. (2011) New regimens to prevent tuberculosis in adults with HIV infection. N Engl J Med 365: 11-20. doi:10.1056/NEJMicm1011576. PubMed: 21732833. 21732833 PMC3407678

[18] South African Department of Health (2013) The South African antiretroviral treatment Guidelines. Available: http://www.doh.gov.za/docs/policy/2013/ART_Treatment_Guidelines_Final_25March2013.pdf.

